# Coat Color‐Associated Transcriptomic Differences of Black and White Hortobágyi Racka Sheep Using the RNA‐Seq Technique

**DOI:** 10.1002/ece3.74397

**Published:** 2026-10-07

**Authors:** Putri Kusuma Astuti, Zoltán Bagi, Lilla Bodrogi, Szilvia Kusza

**Affiliations:** ^1^ Centre for Agricultural Genomics and Biotechnology University of Debrecen Debrecen Hungary; ^2^ Agribiotechnology and Precision Breeding for Food Security National Laboratory, Department of Animal Biotechnology, Institute of Genetics and Biotechnology Hungarian University of Agriculture and Life Sciences Gödöllő Hungary

**Keywords:** coat color, *Ovis aries*, pigmentation, RNA seq, transcriptomics

## Abstract

Coat color is an important visible phenotype in sheep and may reflect underlying differences in pigmentation‐related molecular regulation. However, the transcriptomic mechanisms underlying black and white coat‐color divergence in Hortobágyi Racka sheep remain insufficiently characterized. To address this, this study employed high‐throughput RNA sequencing to analyze the skin transcriptome profiles of black‐ and white‐coated Hortobágyi Racka sheep and identified functional pathways associated with coat‐color difference. We identified 108 DEGs; 25 downregulated and 83 upregulated (FC > 1.5, *p* < 0.05) in black‐coated sheep compared with white‐coated sheep, with the most significant genes including *TYRP1, PMEL, TRPM1, MLANA, SLC24A4, SLC45A2, DCT, OCA2,* and *DDC* (*p* adj < 0.05). Overall, the GO, KEGG, and PPI analyses consistently indicated that coat‐color divergence in Hortobágyi Racka sheep was primarily associated with melanogenesis, melanosome biology, and tyrosine metabolism, while also suggesting possible links with broader cellular process related to redox balance, energy metabolism, and stress responses. However, these broader adaptive interpretations should be considered hypothesis‐generating and require further validation using larger cohorts, phenotypic measurements, and functional assays. Our study provides a transcriptomic framework connecting coat‐color phenotypes to pigmentation regulatory networks and provides a foundation for investigating the possible adaptive significance of coat‐color variation in sheep.

## Graphical abstract

We compared skin transcriptomes of black‐ and white‐coated Hortobágyi Racka sheep using RNA‐seq to identify coat color‐associated molecular differences. Black‐coated sheep showed coordinated upregulation of melanogenesis and melanosome‐related genes together with pathways linked to redox balance, mitochondrial metabolism, and thermogenesis. These findings suggest that pigmentation‐associated transcriptional programs may extend beyond coat color and contribute to cellular processes potentially relevant to environmental adaptation.

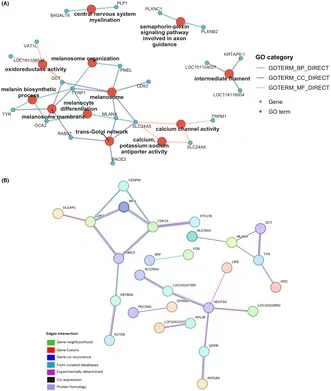

## 1 Introduction

Melanin synthesis, which underlies coat color phenotypes, is governed by a complex network of genes and regulated through the integration of multiple signaling and metabolic pathways (Zhang et al. 2024). Beyond pigmentation alone, melanin has additional protective roles, such as absorbing ultraviolet (UV) radiation, mitigating UV‐induced skin cell damage, scavenging free radicals, and regulating photosensitivity. The proportion and distribution of eumelanin and pheomelanin determine the overall coloration of the coat in ruminants; higher eumelanin content produces darker color, whereas higher pheomelanin content results in lighter color (Díaz et al. 2024). Both pigments derive from dopaquinone, but pheomelanin synthesis is more antioxidant‐intensive; it consumes more glutathione/cysteine (Galván and Solano 2015), which may potentially reduce antioxidant capacity and compromise resilience to environmental stress.

Coat color in livestock has drawn significant interest in relation to heat stress adaptation (Zhang et al. 2024; Leite et al. 2020) amid increasing heat‐stress risk under global climate change. Excessive exposure to UV radiation leads to high reactive oxygen species (ROS) production, which can rapidly exhaust antioxidant capacity and challenge homeostatic regulation (Maibam et al. 2017). This has led to interest in coat pigmentation as a potential adaptive trait, particularly given the unresolved debate on the redox costs of melanogenesis. Current evidence does not support a universal “best” coat color for climate resilience: proposed advantages of lighter versus darker coat vary across studies and settings. For example, Al‐Haidary et al. (2021) suggested that the white coat color is not necessarily a crucial trait for heat tolerance in grazing sheep under hot–arid conditions, as rectal temperature was significantly higher in white‐coated Naemi sheep than in black‐coated Najmi sheep (*p* < 0.05), whereas skin temperature, packed cell volume (PCV), and plasma albumin did not differ between coat colors (*p* > 0.05). In contrast, El‐Sherbiny et al. (2023) showed that under prolonged heat stress, white‐coated and mixed white‐black‐coated bucks showed the highest adaptability, and a 40%–50% black proportion within a white coat did not compromise heat resilience, with deterioration in oxidative‐stress markers observed mainly in darker groups. Breed history and correlated traits, such as hair/wool characteristics, also shape thermal and UV responses (De et al. 2024). Overall, adaptation is inherently context‐dependent, being shaped by the specific environmental pressures and biological constraints experienced by a population.

The Hortobágyi Racka (*Ovis aries strepsiceros hungaricus*), a unique Hungarian national breed recognized for its distinct spiral V‐shape horns, is often used in crossbreeding because of its reported environmental hardiness, particularly its ability to withstand extreme summer and winter conditions. This breed presents two predominant coat colors—uniformly white and uniformly black (Ramírez‐Díaz et al. 2025; Ciani et al. 2020)—making it particularly suitable for coat pigmentation study. Moreover, Zsolnai et al. (2020) reported that the two phenotypes of this breed differ in genetic signatures potentially related to stress adaptation, including putative selection signals at SNPs within *HTR5A* based on 50 K SNPs genotyping.

This study aims to analyze coat color‐associated transcriptome differences in the skin of Hortobágyi Racka sheep with black and white coat colors using high‐throughput RNA sequencing (RNA seq) to characterize the molecular pathways underlying pigmentation differences. In addition, exploring their potential links to cellular stress adaptation by examining the transcriptomic relationship between coat color, pigmentation pathways, and stress‐related molecular signature. This study provides new insights into the molecular basis of coat‐color variation and generates hypotheses for future studies on sheep environmental adaptation.

## 2 Material and Method

### 2.1 Sample Collection

This study used a total of 10 Hortobágyi Racka ewes (white coat = 5, black coat = 5) with a similar age (1.5 to 2 years) and body weight (50 to 60 kg). All animals were raised under identical environmental circumstances and feeding management at Hortobágyi Nonprofit Kft. Sampling occurred at an abattoir in Tomajmonostora, Kiskunhegyes, Jász‐Nagykun‐Szolnok, in the Northern Great Plain region of Hungary (coordinates: 47°25′56.8″ N, 20°42′33.0″ E). Samples were taken in May 2023 with average temperature during the sampling time (09.00–13.00) ranging between 17.1°C–22.5°C. Skin samples were collected from the posterior cervical region of the animal immediately following the slaughtering process (maximum of 1 h postmortem) and were preserved in RNAlater solution (Thermo Fisher Scientific in Waltham, MA, USA) to be stored temporarily in an ice box with 4°C during the transportation to the laboratory and subsequently stored in a freezer at −70°C until RNA isolation.

### 2.2 RNA Extraction, Library Preparation and Sequencing

Total RNA was extracted from skin sample with Direct‐zolTM‐96 MagBead RNA Kit (Zymo Research, Orange, CA, USA) according to the manufacturer's protocol. RNA libraries were generated using the QuantSeq 3′ mRNA‐Seq Library Prep Kit FWD for Illumina (Lexogen, Vienna, Austria). Briefly, 100 ng total RNA was used as input for first strand cDNA generation using oligo(d) to generate first‐strand cDNA, and second‐strand synthesis was initiated by random priming to produce strand‐specific 3′‐end libraries. Double‐stranded cDNA libraries were purified using magnetic beads, PCR‐amplified, and indexed according to the kit instructions prior to sequencing. All libraries were assessed on the Agilent 4200 TapeStation (Agilent Technologies, Palo Alto, CA, USA) to examine if adapter dimers formed during PCR. The QuantSeq libraries were sequenced using the Illumina NovaSeq 6000 platform (Illumina, San Diego, CA, USA) with 2 × 151 run configuration. As the QuantSeq 3′ mRNA‐Seq Library Prep Kit FWD is optimized for single‐end sequencing using Read 1 (recommended read length ~75 bp), paired‐end sequencing is technically possible but not recommended. However, because multiple library types were pooled within the same Illumina run, all samples were sequenced in a 2 × 151 configuration; therefore, the QuantSeq data were subsequently processed by retaining Read 1 only and trimming to 75 bp.

### 2.3 Bioinformatics Analysis

Binary Base Call (BCL) files generated by the sequencing instrument were base called, demultiplexed and translated into FASTQ files using bcl2fastq v2.20.0.422 (Illumina Inc.). Reads were subsequently trimmed to 1 × 75 bp (minimum length: 40 bp) and quality‐trimmed to Q30 using the Phred algorithm with BBDuk from the BBTools suite v38.86 (Bushnell et al. 2017).

The processed reads were aligned against the domestic sheep ( *Ovis aries* , Rambouillet breed, OAR_USU_Benz2616 strain) reference genome downloaded from NCBI (ARS‐UI_Ramb v3.0 assembly; July 20, 2023; RefSeq: GCF_016772045.2) using STAR v2.7.6a (Dobin et al. 2013). The number of reads aligned within each gene was counted using HTSeq v0.12.4 Python library (Anders et al. 2015). Gene count data were normalized using the trimmed mean of *M*‐values (TMM) method of the edgeR v3.28 (R v3.6.0, Bioconductor v3.9) and transformed using the voom approach in limma v3.42 to model the mean–variance relationship and generate precision weights for downstream linear modeling (Ritchie et al. 2015). Differential expression between coat‐color groups was assessed in limma v3.42 using a weighted linear model with coat color as the main effect (design: ~coat_color; contrast: black vs. white). No additional covariates were included. Because RNA‐seq data are discrete counts and exhibit mean–variance dependence, normality and homogeneity‐of‐variance tests were not applied to raw expression values. Instead, the voom transformation in limma v3.42 was used to estimate the mean–variance trend and apply precision weights, which addresses heteroscedasticity typical of RNA‐seq data. Sample‐level quality and clustering were additionally assessed by principal component analyses (PCA). Significance was defined using a combined threshold of fold change (FC) > 1.5 and *p* < 0.05 from limma's moderated *t*‐test. Benjamini‐Hochberg FDR was applied for multiple‐testing correction (*p* adj < 0.05). For exploratory data analysis and visualization, normalized expression values were also represented as transcripts per million (TPM).

### 2.4 Function Enrichment Analysis

The DEGs (all, upregulated, and downregulated) underwent gene ontology (GO) and Kyoto Encyclopedia of Genes and Genomes (KEGG) pathway enrichment analysis using the Database for Annotation, Visualization, and Integrated Discovery (DAVID) (https://david.ncifcrf.gov/tools.jsp) (Dennis et al. 2003) website for three primary GO categories: biological process (BP), cellular component (CC), and molecular function (MF). Using the background genome of *Ovis aries*, GO term or KEGG pathways were considered significant if their corrected *p* < 0.05. Visualizations were constructed using the ggplot2 package v3.3.6 (Wickham 2016) in R v3.6.0. Further, a GO gene‐term network was constructed and visualization were performed using igraph v2.3.3, ggraph v2.2.2, and ggplot2 v3.3.6 (Dennis et al. 2003; Csárdi and Nepusz 2006; Wickham 2016).

Protein–protein interaction (PPI) networks for all DEGs were built in STRING Database (https://string‐db.org) with *Ovis aries* as background organism and TRING evidence channels with a minimum interaction score of 0.25, disconnected nodes were excluded, and interaction networks were constructed using the following evidence channels: experiments, curated databases, co‐expression, genomic neighborhood, gene fusion, and phylogenetic co‐occurrence.

### 2.5 Validation of Differentially Expressed Genes

A total of 10 genes (*IRF4, PMEL, GPNMB, TYRP1, PLXNC1, VNN1, TYR, PLP1, SLC24A5*, and *TRPM1*) were chosen from the 108 DEGs list to validate the gene expression in skin tissue of black‐coated and white‐coated Hortobágyi Racka sheep using qRT‐PCR. The selected genes were prioritized based on their relatively high expression levels, differential expression, and biological relevance to the main enriched functional categories, including pigmentation, immune or stress‐related processes, and metabolic or signaling pathways. Primer Express v3.0.1 software (Applied Biosystems, Foster City, CA, USA) was used to design the primers and was validated using Primer Blast from NCBI (Ye et al. 2012) (Table 1).

**TABLE 1:** Primers details of 10 target genes and one housekeeping gene for qRT‐PCR validation.

Target gene	GenBank accession	Primer	Amplified product (bp)
*IRF4*	XM_060403818.1	F: TATCAGTGTCCCGTGACCTTC R: TGTGACCTGGCAACCATTTTC	78
*PMEL*	XM_004006599.6	F: GCCCTTCTGGCCCTTTATCT R: ACTTGCCAGTATTGGTCCCAG	73
*GPNMB*	XM_004007790.6	F: ACCACCCCTTCTTTGGTAACTG R: CATCAGGAATCTCCCTCAGCTC	61
*TYRP1*	XM_042242266.2	F: AAGTTCAATGGCCAGGTCGG R: TATCCAGAGCCTGGACAAAGC	72
*PLXNC1*	XM_015094600.3	F: TGGAGCTAAAAAGTGCCCTCAA R: CCACCGTCACTGTCGTCTTAT	68
*VNN1*	XM_004011337.6	F: GAGAAGGATAGTGCACCAAATGC R: TGAGCATCACAAATACCTTAGCG	69
*TYR*	NM_001130027.1	F: CAGGTTCCATGGATAAAGCTGC R: GCGGACTAGCAAATCCTTCCA	66
*PLP1*	XM_004022464.6	F: CCAGCAAGACCTCTGCAAGTA R: TTCCATGGGAGAACACCATACA	70
*SLC24A5*	NM_001252181.1	F: CTCCCAAGGGCTACAGGAAATAG R: TCGTGAAAAACCCCTCAGGAAA	76
*TRPM1*	XM_060401392.1	F: ACTGCCCTGCTGAAAGGAAC R: ATGTCCACACGGTTCCAAGC	77
*GAPDH*	NM_001190390.1	F: CATCCCTGCTTCTACTGGCG R: CCAGTGAGCTTCCCGTTGAG	72

The qPCRBIO cDNA Synthesis Kit (PCR Biosystems, London, UK) was used. The template consisted of cDNA equivalent to 5 ng of the initial total RNA. The Roche Light Cycler96 was utilized for qPCR with a 3‐min denaturation step, followed by 50 cycles of 95°C for 15 s, 62°C for 20 s, and 72°C for 15 s. Each run underwent high resolution melting analysis. PowerUpTM SYBR! Green Master Mix (Applied Biosystem, Foster City, CA, USA) was used with each sample was subjected to qRT‐PCR in triplicate.

In order to conduct relative gene expression investigations, the amplification of a housekeeping gene, *GAPDH*, was performed in conjunction with the 10 target genes. Changes in gene expression (CT value) were calculated using the 2^−ΔΔCt^ (DDCT) method, the fold changes then analyzed with independent sample *t*‐test.

## 3 Results

### 3.1 Aligned RNA Seq Data

A total of 54,017,887 reads were generated from the Illumina NovaSeq 6000 platform sequencer from the nine animals as one of the black‐coated animals failed to be sequenced. This read was successfully annotated to 21,328 genes from *Ovies aries* genome (ARS‐UI_Ramb v30). Given the QuantSeq 3′ library structure and the use of single‐end 75 bp reads, elevated multi‐mapping was expected, particularly for repetitive regions and paralogous skin‐expressed gene families. Alignment metrics were summarized per sample (Table 2). During gene‐level quantification with HTSeq, ambiguously assigned reads were not counted toward gene totals, reducing potential inflation from multi‐mapped reads.

**TABLE 2:** The summary of alignment statistic of black‐ (*n* = 4) and white‐ (*n* = 5) coated Hortobágyi Racka sheep.

Sample ID	Total reads	Uniquely mapped	% uniquely mapped	Multiply mapped	% multiply mapped
White 1 (10.5)	7,164,854	4,491,865	62.69	2,580,968	36.02
White 2 (10.14)	6,460,274	4,140,255	64.09	2,218,596	34.34
White 3 (10.13)	4,241,093	2,144,268	50.56	2,026,868	47.79
White 4 (10.10)	5,393,951	3,094,769	57.37	2,243,251	41.59
White 5 (10.3)	7,406,806	4,399,639	59.40	2,931,958	39.58
Black 1 (5.1)	5,478,963	2,937,849	53.62	2,421,147	44.19
Black 2 (5.12)	6,209,220	3,770,964	60.73	2,377,245	38.29
Black 3 (11.1)	4,085,081	2,312,890	56.62	1,702,935	41.69
Black 4 (11.8)	7,577,645	5,155,140	68.03	2,344,402	30.94

### 3.2 Differentially Expressed Genes Between Black‐Coated and White‐Coated Hortobágyi Racka Sheep

Based on the threshold criteria FC > 1.5 and *p* < 0.05, a total of 108 genes were identified as differentially expressed between black‐ and white‐coated Hortobágyi Racka sheep (Supplementary File 1), of which 25 genes were downregulated, while 83 genes were upregulated (Figure 1). Functionally, these downregulated DEGs span epidermal differentiation and barrier (*KRTAP6‐1, KRT1, LGALS7, SPTSSB*), tissue organization (*FREM1, OPCML, SUSD6, PLXNB2, ARHGAP18*), stress‐responsive and regulatory signaling (*FOS, STK17B, KCTD6, KHDRBS2*), and ion‐channel‐associated signaling (*KCNJ6, SNAP25, CA10*), alongside some uncharacterized loci. Overall, the upregulated DEG set was dominated by a conserved pigmentation network, including melanogenesis enzymes (*TYR, TYRP1, DCT*), melanosome structure (*PMEL, MLANA, GPNMB, TRPM1*), melanosomal transport (*OCA2, SLC45A2, SLC24A5, SLC24A4*), and upstream melanocyte regulatory (*PAX3, IRF4, RAB32*), which was consistent with coordinated regulation of pigment synthesis and deposition. Additional upregulated DEGs implicated broader cell signaling and stress response (*NR4A3, ATF5, PPM1E, PRKG2*), membrane trafficking and channel handling (*SNAP91, RANGRF*), and lipid/redox and epithelial‐barrier–relevant metabolism (*LIPE, AKR1B1, VNN1, B4GALT6, B3GALNT1, DDC*), alongside tissue‐organization pathways (*PLXNC1, LYPD1, GPRIN3, DLGAP1, CSRP2, PAG3, CDK15*) and myelin‐associated transcripts (*PLP1, NNAT*). The multi‐testing Benjamini‐Hochberg FDR showed that *TYRP1, PMEL, TRPM1, MLANA, SLC24A4, SLC45A2, DCT, OCA2*, and *DDC* were significantly upregulated (*p* adj < 0.05, Figure S1).

**FIGURE 1:**
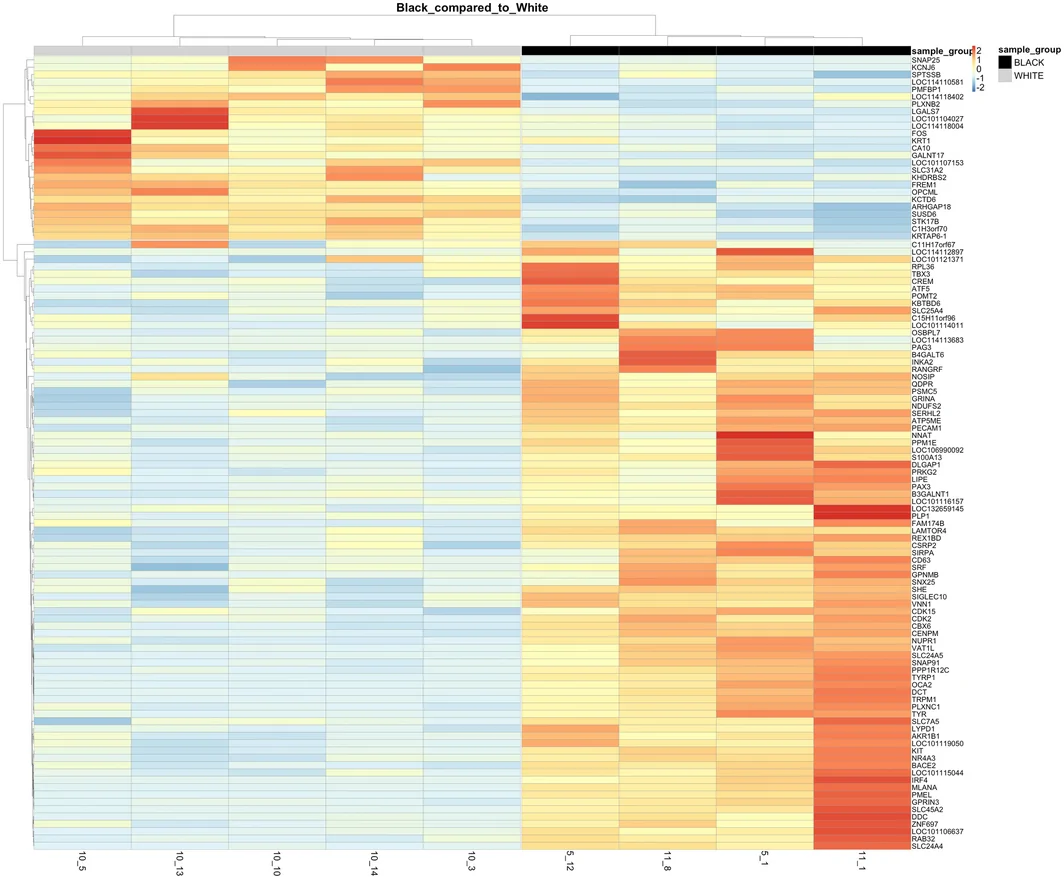
The heat map of the total read per million (TPM) value of 108 differentially expressed genes (*p* < 0.05) between the black‐ (*n* = 4) and white‐ (*n* = 5) coated Hortobágyi Racka sheep's skin. Genes and samples were hierarchically clustered based on overall expression‐profile similarity using Pearson correlation. Red indicates upregulated expression, blue shows downregulated expression, and color intensity reflects the expression level.

### 3.3 Enrichment and Pathway Analysis

The combined analyses of upregulated and downregulated DEGs yielded 12 significant functional annotations (*p* < 0.05). Independent analysis of the upregulated (*n* = 83) and downregulated (*n* = 25) gene sets resulted in 11 and 2 significant functional annotations (*p* < 0.05), respectively (Figure 2A). The result of the all DEGs analysis omitted GO‐BP:0031424~Keratinization and GO‐MF:0004190~Aspartic‐type endopeptidase activity, while the separated up/downregulated DEGs analysis excluded GO‐BP:1902287~Semaphorin‐plexin signaling pathway involved in axon guidance.

**FIGURE 2:**
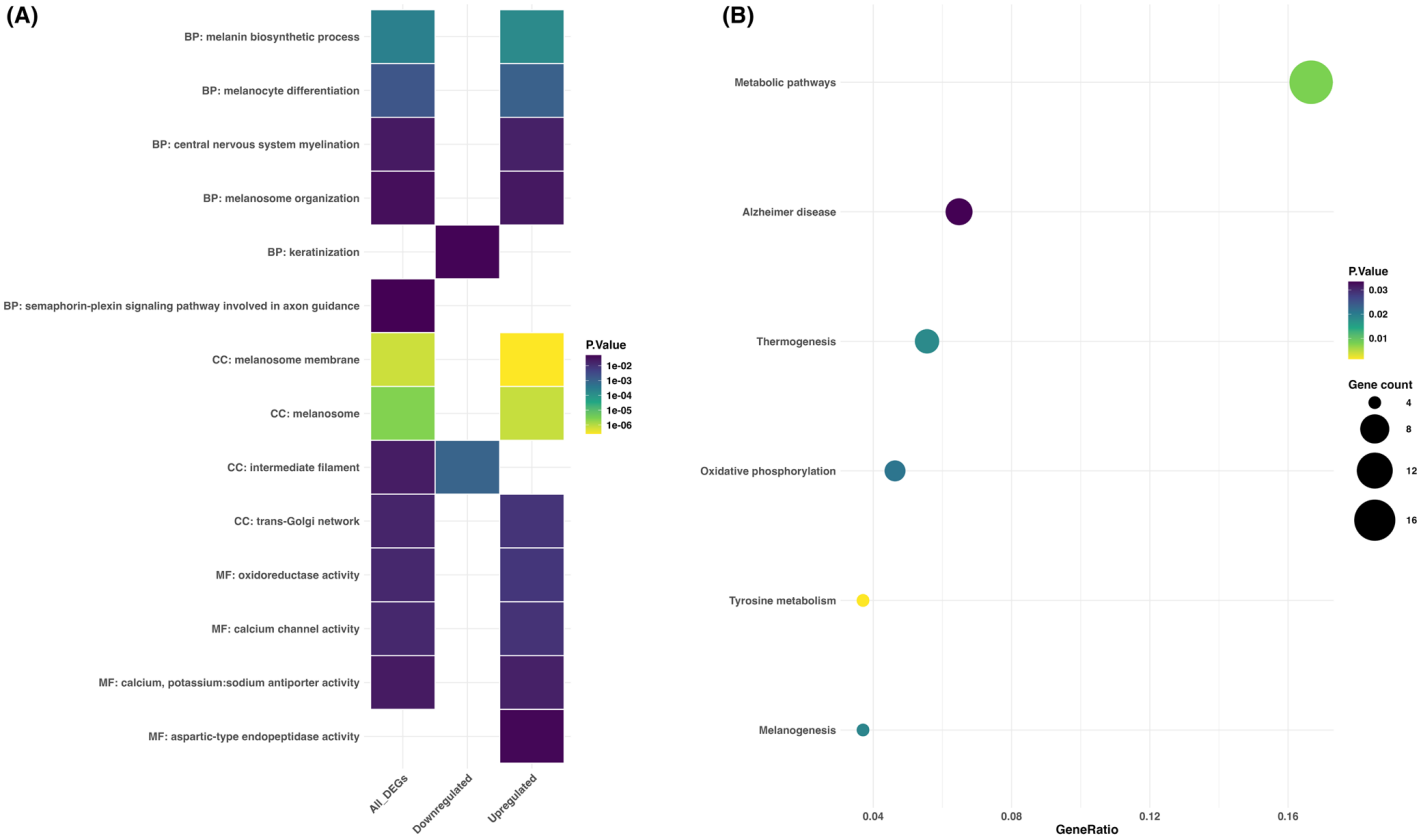
(A) Significantly enriched GO functional annotations (*p* < 0.05) derived from all, upregulated, and downregulated DEGs, and (B) bubble plot for the KEGG pathway enrichment from all 108 DEGs between black‐coated and white‐coated Hortobágyi Racka sheep.

Similar differences were observed in the KEGG pathways analysis, which identified six significantly enriched pathways (*p* < 0.05) when all DEGs were evaluated (Figure 2B), including oxidative phosphorylation, tyrosine metabolism, metabolic pathways, thermogenesis, melanogenesis, and Alzheimer's disease. When analyzed separately, the identical set of pathways was enriched for the upregulated except for the oxidative phosphorylation pathway, while the downregulated DEGs did not yield any significant enriched pathways.

The GO gene–term network constructed from all DEGs (Figure 3A) showed a clear functional organization centered on pigmentation and melanosome‐related processes with some pigmentation‐associated genes (i.e., *TYR, TYRP1, OCA2, DCT, PMEL, MLANA, SLC24A5*, and *RAB32*), contributed to multiple connected GO terms. In addition, the network showed smaller functional modules related to ion transport, including calcium channel activity and calcium/potassium:sodium antiporter activity (i.e., *TRPM1, SLC24A4*, and *SLC24A5*).

**FIGURE 3:**
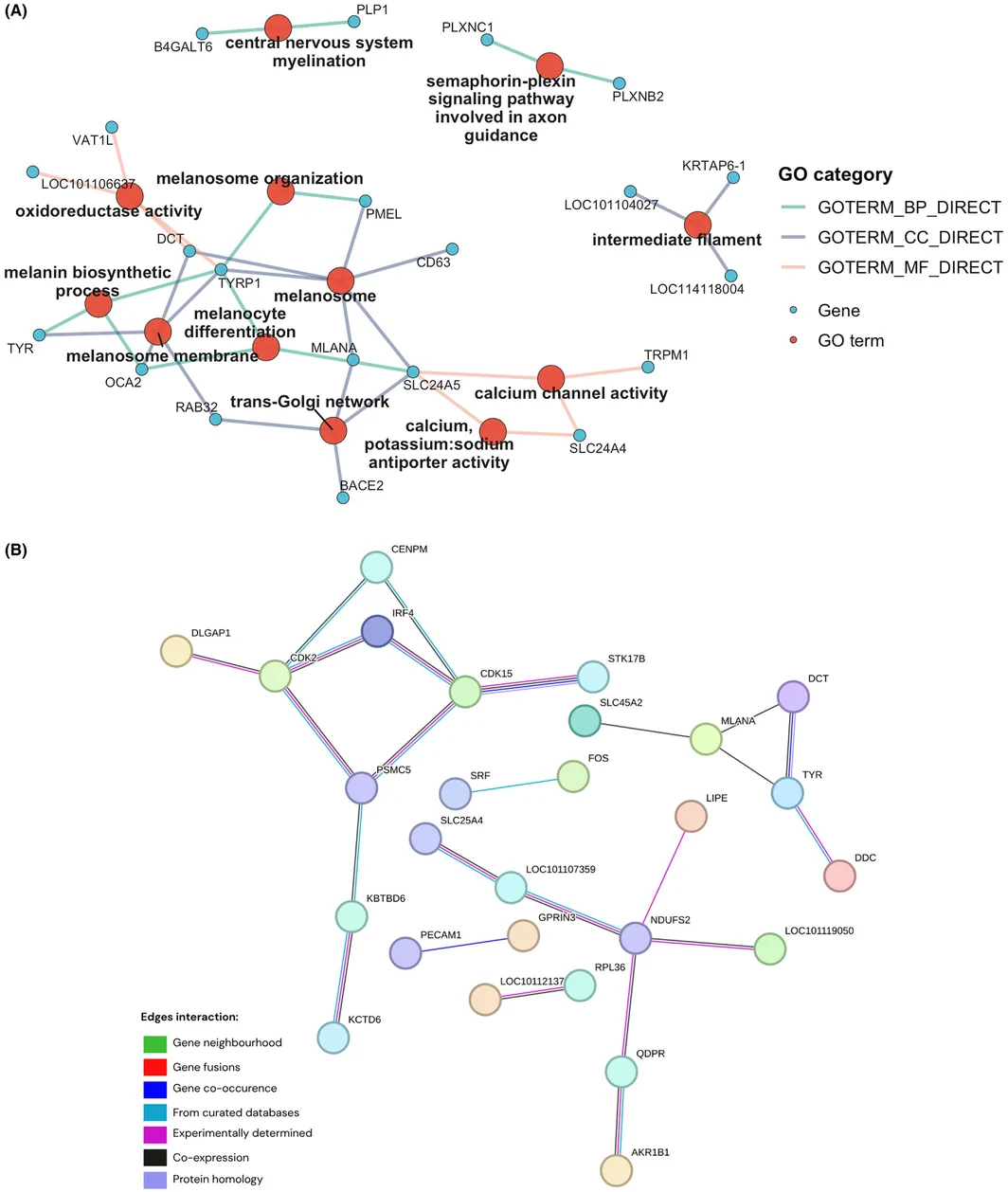
Interaction network using all DEGs between black and white‐coated Hortobágyi Racka sheep based on (A) GO gene‐term network and (B) protein–protein interaction based on STRING database, with odes outside the network are removed for clarity, and edge color indicates interaction type.

Additionally, the STRING database was used to visualize the PPIs of the all DEGs in the black‐coated Hortobágyi Racka sheep (Figure 3B) and identified a coherent melanogenesis interaction module cantered on *TYR–DCT–MLANA*, with a link to melanosome transport via *SLC45A2* and *DDC*. Additional connected components were enriched for mitochondrial/redox metabolism (*NDUFS2–SLC25A4–QDPR–AKR1B1, LIPE, LOC101107359, and LOC101119050*) and cell cycle/proteostasis signaling (*CDK15, CDK2, CENPM, PSMC5*, with *IRF4*, *STK17B, DLGAP1, KBTBD6*, and *KCTD6*). The presence of small modules (i.e., *FOS–SRF, PECAM1–GPRIN3*, and *LOC10112137–RPL36*) suggests broader responsive transcriptional regulation.

### 3.4 RNA‐Seq Data Validation With RT‐qPCR

To validate the RNA‐Seq result, RT‐qPCR was performed to measure the expression of 10 randomly selected genes from the DEG list in the skin of black and white Hortobágyi Racka sheep, including those involved in skin pigmentation (*TYRP1, SLC24A5, PMEL*, and *TYR*), immunological functions (*IRF4, PLXNC1*, and *GPNMB*), metabolic pathways (*TRPM1, VNN1*, and *PLP1*). Consistent with the transcriptome sequencing data, RT‐qPCR results showed that 8 out of 10 selected genes were significantly more highly expressed in black sheep skin compared to white sheep skin (*p* < 0.05). The most significant variation in gene expression between the two groups was observed for *TYRP1* (Figure 4).

**FIGURE 4:**
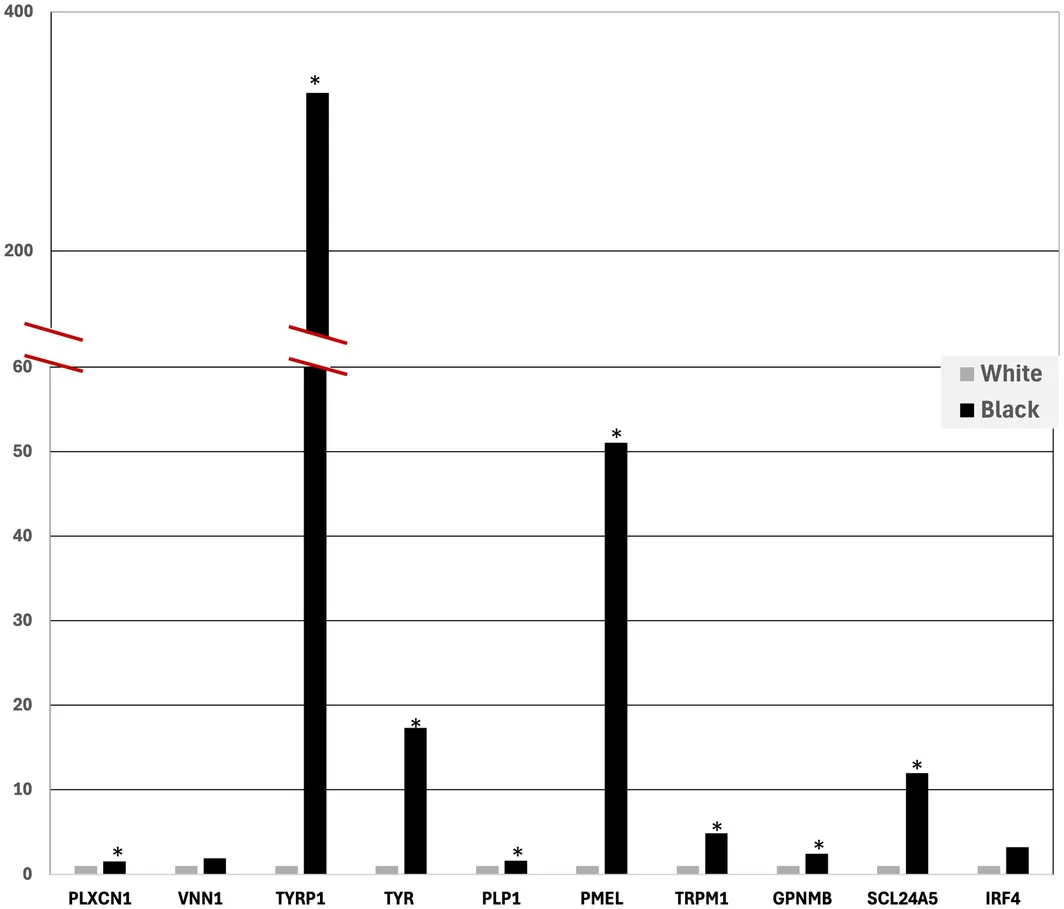
RT‐qPCR validation of 10 differentially expressed genes in white‐ and black‐ coated Hortobágyi Racka sheep skin. The expression level of target genes was normalized relative to that of the *GAPDH* gene. The bars in each panel represent the mean and *p < 0.05.

## 4 Discussion

The aim of this study was to compare the skin transcriptome of black‐coated and white‐coated Hortobágyi Racka sheep using RNA‐seq analysis. Although based on a modest sample number (*n* = 9; 4 vs. 5), our study is comparable to published skin transcriptome analysis by Vasu et al. (2024) in Changthangi versus Muzzafarnagri sheep. In addition, our research design limits confounding from breed‐level genetic structure and supports more targeted detection of coat‐color–associated expression differences by contrasting different coat color phenotypes within the same breed.

The number of DEGs (FC > 1.5, *p* < 0.05) identified in our study was lower than in previous skin transcriptomic studies in goats and sheep reported by Xiong et al. (2019) and Yao et al. (2019), but slightly higher than a study in Laiwu Black goat and Lubei White goat by Peng et al. (2018).

Our findings indicated a higher number of upregulated genes (*n* = 83) than the downregulated ones (*n* = 25) when compared between black to white‐coated Hortobágyi Racka sheep, with the transcriptome of black‐coated sheep distinguished by overexpression of genes associated with pigmentation and cellular processes of melanogenesis. These genes have also been identified in prior pigmentation studies in sheep (Yao et al. 2019; Shi et al. 2021; Zhang et al. 2023), all of which have shown a clear correlation between lower expression of melanin‐related genes and a lighter coat and/or skin phenotype.

Despite the pigmentation related roles of the significantly upregulated genes (*p* adj < 0.05), some of these genes have been reported in previous research to be involved in livestock's adaptation and immunity. In Dorper's liver transcriptome investigation by Wang et al. (2020), *TRPM1* was claimed to possibly perform DNA repair and promote protein stability with increasing calcium ion entry for the positive reaction to heat stress. While according to Ali et al. (2023), *TYRP1* regulates animals' oxidative stress, facilitates cellular proliferation, and promotes adaptation to extreme conditions. Comparative investigations have demonstrated that mutations in *TYRP1* can modify protein structure and function, affect enzyme function and melanin biosynthesis pathways, hence facilitating adaptation in indigenous sheep and survival in various environments. *SLC* genes are essential for maintaining the homeostasis of the central nervous system through the selective and regulated transport of nutrients, hormones, electrolytes, metal ions, and metabolic wastes. This gene family also regulates mTOR, thereby contributing to protein synthesis and amino acid absorption, and has been associated with pro‐inflammatory factors (Gaowa et al. 2021; Li et al. 2024). Zhang et al. (2024) further reported that *TYRP1, DCT, PMEL, MLANA*, and *SLC45A2* were significantly associated with metabolite prostaglandins which function in wound healing and inflammatory responses.

The GO enrichment analysis and the GO gene‐term network showed enriched MFs including calcium, potassium:sodium antiporter activity and calcium channel activity, which may reflect intensified melanocyte/melanosome physiology, because melanogenesis is tightly regulated by organellar ion homeostasis (e.g., *SLC24A5/NCKX5*) and melanocyte ion‐channel signaling (e.g., *TRPM1*) that track pigment‐producing states (Ginger et al. 2008). Enrichment of oxidoreductase activity is also biologically plausible given that pigment synthesis and related pathways can impose substantial redox demands and/or be associated with ROS generation, making redox‐buffering and detoxification processes relevant co‐signatures of active melanogenesis (Lu et al. 2021).

The STRING results further suggest a possible network‐level relationship between melanogenesis and cellular stress‐response pathways, although not providing direct functional evidence of stress adaptation. While melanin enhances photoprotection, pigment synthesis involves redox‐active reactions that can increase oxidative pressure; *DCT* may contribute to limiting intermediate toxicity (Dolinska et al. 2022; Wang et al. 2024). The accompanying mitochondrial and proteostasis‐related components may reflect broader bioenergetic and protein‐quality‐control adjustments, while *SLC45A2* may link melanosome pH regulation with proteasome‐associated control (Le et al. 2020).

Similarly, the KEGG results resolve into a clear pigmentation core (melanogenesis and tyrosine metabolism) together with a broader metabolic/mitochondrial adaptation axis (metabolic, oxidative phosphorylation and thermogenesis pathways). These pathways involved mitochondrial genes (*NDUFS2, ATP5ME, SLC25A4*) and lipid/signaling components (*LIPE, PRKG2*). However, these enrichment results should be interpreted as evidence of coordinated transcriptional differences in genes annotated to mitochondrial and metabolic pathways, rather than as direct stress physiology and adaptation evidence. This may suggest that the black‐white pigmentation contrast co‐occurs with transcriptional differences in cellular bioenergetics and stress physiology, a pattern that is biologically plausible given the energetic/redox demands of active skin and hair follicle programs and the central role of mitochondrial pathways in thermal adaptation (Jiao et al. 2021; Zhu et al. 2025). Alzheimer disease KEGG pathway enrichment should not be interpreted solely as evidence of neurodegenerative pathology. In this context, the enrichment likely reflects the inclusion of broad cellular stress programs that can be activated in many tissues, including Ca^2+^/mitochondrial electron transport and stress‐response components that overlap with core bioenergetic pathways and pigmentation (Rochin et al. 2013; Popugaeva et al. 2018). Although Davies et al. (2022) reported that sheep can exhibit Aβ and phosphorylated tau in the brain, supporting the presence of Alzheimer disease‐linked molecular modules in sheep.

Biologically, Koike and Yamasaki (2020) highlighted that melanocytes release cytokines that affect immunological responses, indicating that they may serve as antigen‐presenting cells. Melanocytes can function as target cells for T lymphocytes by processing and presenting phagocytosed antigens. They express ICAM‐1, an essential component of immune cell interactions, and CD40, which is typically present in mature dendritic cells and facilitates adaptive immunity. Melanocytes with higher melanin content might suppress inflammation more effectively due to the immunosuppressive effects of L‐DOPA and its products. Meanwhile, melanocytes with lower melanin content may contribute more actively to cytokine‐mediated immune signaling, potentially promoting a stronger immune response (Fu et al. 2020). This suggests that individuals with reduced melanin levels may exhibit distinct immunological profiles, which are not necessarily indicative of superior immunity, but rather reflect differential immune activity due to diminished melanin‐associated immunosuppression.

Beyond its connection to immunity, the relationship between pigmentation and hormones involved in stress response in livestock has been the subject of some prior research. Cortisol is the main mediator of the hypothalamic–pituitary–adrenal (HPA) axis, which is activated in response to stress. The biochemistry of pigment regulation and cortisol regulation partly overlaps, involving shared hormone and receptor families, specifically proopiomelanocortin (POMC). It has been proposed that the availability of POMC for cortisol synthesis may be reduced if more of it is utilized for pigmentation. On the other hand, variations in receptor sensitivity, such as those involving melanocortin receptors, may impact both pigmentation and the response to cortisol. A decreased cortisol response may be adaptive if it aids in avoiding immune suppression and other long‐term consequences of stress, according to the adaptive trade‐offs hypothesis (Nejad et al. 2016; Fernandes et al. 2023).

Eumelanin, the pigment responsible for darker coloration, possesses stronger antioxidant properties and is more effective at blocking UV radiation than pheomelanin. Its increased production may inhibit the formation of ROS and reduce oxidative damage during thermal stress, potentially contributing to improved protection in darker pigmented skin. As Maibam et al. (2017) suggested, most breeds originating from tropical regions have dark coat color, as observed in Zebu cattle. Their environments may enhance the expression of color‐related genes in skin cells, resulting in improved skin protective capacity and a reduction in oxidative damage under environmental heat stress. In addition, a recent *PMEL* gene editing study in Holstein cattle by Wei et al. (2023) demonstrated a significant decrease in the capacity of color‐diluted calves to absorb heat and light compared with control calves with darker coats. Therefore, although light‐colored coats may provide a physical advantage by reflecting more solar radiation and reduced absorption, the upregulation of pigmentation‐related genes such as *TRPM1* and *TYRP1* in black‐coated sheep may reflect enhanced melanocyte activity, melanosome function, ion homeostasis, and pigmentation‐associated redox regulation. This does not necessarily indicate that black‐coated animals are more heat tolerant, but suggests that darker pigmentation may involve distinct cellular mechanisms that could help manage the higher energetic or oxidative demands associated with active pigmentation. Given that pigmentation can also be influenced by environmental stressors, it is plausible that skin pigmentation could contribute to evolutionary adaptation in response to ongoing climate change. Thus, these findings suggest a possible link between darker coat color and environmental resilience. The characteristics and structure of wool or hair also likely contribute to their thermoregulatory capacity. Lower‐pigmented skin has been shown to produce higher levels of oxidative stress compared with highly pigmented skin under conditions of heat stress.

Several limitations should be considered when interpreting these findings. The sample size was limited, comprising four black‐coated and five white‐coated animals, which may reduce statistical power, increase uncertainty in fold‐change and variance estimates, and limit the detection of genes with moderate expression difference. Nevertheless, the coordinated expression of key pigmentation genes and melanosome‐related pathways supports the biological consistency of the main coat‐color‐associated transcriptomic signal. In addition, the study did not include direct phenotypic association analyses or physical measurements related to adaptive traits such as heat tolerance, UV response, oxidative‐stress status, skin temperature, or thermoregulatory capacity. Although RT‐qPCR validated selected transcriptional changes, the proposed links between pigmentation and adaptation were not validated at the protein, biochemical, or physiological levels. Therefore, the observed links between pigmentation, mitochondrial function, redox metabolism, stress‐response and adaptation pathways should be interpreted as hypothesis‐generating rather than causal. The absence of protein‐level assays, oxidative‐stress biomarker assessments, mitochondrial function assays, and controlled heat challenge experiments further limit the strength of the conclusions. Larger studies integrating transcriptomic, phenotypic, environmental, and functional data are needed to validate these findings.

## 5 Conclusion

The transcriptomic differences between black‐ and white‐coated Hortobágyi Racka sheep were dominated by a strong pigmentation signature, involving key melanogenesis, melanosome, and tyrosine‐metabolism genes such as *TYRP1, PMEL, TRPM1, MLANA, SLC24A4, SLC45A2, DCT, OCA2*, and *DDC*. Further, the functional enrichment, GO gene‐term network, and PPI network analyses suggest that coat‐color‐associated expression differences extend beyond pigment production alone; the enrichment of ion transport, oxidoreductase activity, mitochondrial metabolism, oxidative phosphorylation, thermogenesis, and proteostasis‐related components indicates that active melanogenesis may be followed by broader changes in redox balance, organelle homeostasis, cellular bioenergetics, and stress‐response physiology. These findings support the hypothesis that sheep's skin pigmentation might be biologically linked to adaptive cellular processes through pleiotropic effects, particularly those related to photoprotection, oxidative stress buffering, and mitochondrial function. Overall, this study provided transcriptomic evidence that coat‐color divergence in Hortobágyi Racka sheep is primarily associated with pigmentation and melanosome‐related gene expression, while the observed enrichment of stress‐related molecular pathways may suggest potential links with cellular cell response. This interpretation remains hypothesis‐generating, offering a basis for future functional validation in larger and environmentally characterized populations.

## Author Contributions

**Putri Kusuma Astuti:** data curation (equal), formal analysis (equal), visualization (equal), writing – original draft (equal), writing – review and editing (equal). **Zoltán Bagi:** data curation (equal), validation (equal), writing – review and editing (equal). **Lilla Bodrogi:** methodology (equal), writing – review and editing (equal). **Szilvia Kusza:** conceptualization (equal), resources (equal), supervision (equal), writing – review and editing (equal).

## Funding

P.K.A.'s study was supported by Tempus Public Foundation within the Stipendium Hungaricum Programme.

## Ethics Statement

The research was authorized by the University of Debrecen's local ethics committee (registration number 19/2023/DEMÁB). All procedures adhered to the European Union's Animal Experimentation Directive (Directive 2010/63/EU) and the ARRIVE standards.

## Conflicts of Interest

The authors declare no conflicts of interest.

## Supporting information


**Figure S1:** ece374397‐sup‐0001‐FigureS1.png.

**Supplementary File 1:** ece374397‐sup‐0002‐Supinfo.xlsx.

## Data Availability

All data generated or analyzed during this study are included in this article and raw data is available on https://doi.org/10.5281/zenodo.20923295.
